# Effect of Blood Purification on Early Response in Children With Hemophagocytic Lymphohistiocytosis

**DOI:** 10.1111/jcmm.70811

**Published:** 2025-11-03

**Authors:** Lihua Yu, Danna Lin, Li Wu, Lulu Huang, Xiaorong Lai, Yajie Zhang, Juan Zi, Jingxin Zhang, Xu Liao, Lichan Liang, Guanmei Zhang, Liucheng Yang, Lihua Yang

**Affiliations:** ^1^ Department of Pediatric Hematology Zhujiang Hospital, Southern Medical University Guangzhou China; ^2^ Department of Pediatric Surgery Zhujiang Hospital, Southern Medical University Guangzhou China

**Keywords:** blood purification, early responses, hemophagocytic lymphohistiocytosis, mediation analysis, paediatric

## Abstract

To evaluate the impact of blood purification (BP) therapy on early treatment response and overall survival (OS) in paediatric haemophagocytic lymphohistiocytosis (HLH). We retrospectively reviewed HLH cases from 2012 to 2022, comparing outcomes among patients treated with the HLH‐94/04 protocol, BP monotherapy, BP combined with HLH‐94/04 (BP + HLH‐94/04), or other regimens. A matched subgroup analysis was conducted in patients with multi‐organ dysfunction syndrome (MODS) to assess survival differences. Cox regression identified prognostic factors, and mediation analysis evaluated the contribution of complete remission (CR) at 4 weeks to OS. A total of 102 patients were included, five with primary HLH. Among them, 53 received HLH‐94/04, 13 received BP monotherapy, 22 received BP + HLH‐94/04, and 14 received other treatments. OS differed by treatment: HLH‐04 (81.3%), HLH‐94 (76.6%), other regimens (47.6%), BP + HLH‐94/04 (23.4%), and BP monotherapy (15.4%) (*p* < 0.001). In MODS patients, survival was 37.5% (HLH‐94/04), 30.8% (BP + HLH‐94/04), and 8.3% (BP monotherapy) (*p* = 0.013). Among partial/non‐responders (PR/NR) at 4 weeks, survival occurred only in the HLH‐94 group (28.6%) (*p* = 0.018); in PR/NR with MODS, only BP + HLH‐94/04 showed survival (18.2%) (*p* = 0.008). CR at 4 weeks, central nervous system involvement, and elevated lactate dehydrogenase were independent predictors of OS (*p* < 0.05). Mediation analysis showed CR contributed 79.88% to OS in BP + HLH‐94/04 and 33.28% in BP monotherapy. BP combined with HLH‐94/04 may improve survival in patients with MODS or poor early response. Early CR at 4 weeks is a key prognostic marker.

**Abbreviations**
ALB: albumin
ALT: alanine transaminase
AST: aspartate transferase
CNS: central nervous system
CR: complete response
DBIL: direct bilirubin
EBV: Epstein–Barr virus
HLH: hemophagocytic lymphohistiocytosis
LDH: lactate dehydrogenase
NR: no response
OS: over survival
PICU: paediatric intensive care unit
PR: partial response
TBIL: total bilirubin
WBC: white blood count

## 1 Introduction

Hemophagocytic lymphohistiocytosis (HLH) is a potentially fatal condition resulting from an exaggerated immunological response to antigens, which triggers uncontrolled activation of immune cells and excessive release of cytokines [1]. The HLH‐94/04 protocols offer a comprehensive therapeutic approach that utilises a multi‐drug cocktail therapy to suppress the hyperinflammatory condition in T cells and macrophages. However, the complexity and heterogeneity of the disease can result in some patients not responding well to this therapy, ultimately leading to fatalities [2].

Extracorporeal blood purification (BP) techniques, such as plasmapheresis and continuous renal replacement therapy, have demonstrated efficacy in eliminating inflammatory mediators and toxins while also providing support to multiple organs in individuals with Epstein–Barr virus (EBV)‐associated hemophagocytic lymphohistiocytosis (EBV‐HLH) [3]. However, evidence supporting the effectiveness of BP in treating HLH remains limited. Early mortality continues to pose a significant challenge during HLH therapy. Therefore, it is essential to determine whether BP can contribute to remission by the 4‐week mark.

In this study, we aimed to evaluate the impact of BP therapy on early treatment response and overall survival (OS) in paediatric HLH. We compared outcomes across different treatment strategies, including HLH‐94 protocol, HLH‐04 protocol, BP monotherapy, and BP combined with HLH‐94/04. Special attention was given to patients with multi‐organ dysfunction syndrome (MODS) and those classified as partial or non‐responders (PR/NR) at 4 weeks, as these subgroups represent high‐risk populations with poor prognoses. Additionally, we used causal mediation analysis to assess whether complete remission (CR) at 4 weeks mediated the relationship between BP‐based treatments and OS, while also identifying key clinical and prognostic factors.

## 2 Patients and Methods

The study involved a retrospective cohort of patients diagnosed with HLH at Zhujiang Hospital, Southern Medical University, from 1 January 2012, to 31 December 2022. The last follow‐up time was 1 May 2023. The Institutional Review Board of Zhujiang Hospital of Southern Medical University approved the study on 1 December 2022 (ID:2023‐KY‐258‐01). The study followed the principles outlined in the Declaration of Helsinki (revised in 2013).

The inclusion criteria for HLH patients include those aged 18 years or younger and those diagnosed with the revised HLH‐04 diagnostic criteria [4]. Patients whose outcomes were not tracked during the survey and those whose HLH was triggered by medical interventions or treatments that activate the immune system were excluded. Demographic, clinical and laboratory data were collected for each patient, including age, gender, family history, fever duration, hepatomegaly, splenomegaly, and lymphadenopathy, laboratory parameters, treatments administered and outcomes. The laboratory diagnostic criteria for soluble CD25 levels (*n* = 28) and natural killer cells (*n* = 16) were only accessible to some patients with additional medical insurance. The HLH‐94 and HLH‐04 protocols were used to treat HLH [5, 6]. Other treatments include corticosteroids, intravenous immunoglobulin, and/or ruxolitinib therapy.

BP monotherapy was given as the sole treatment for HLH in patients with multiple organ dysfunction who refused HLH‐94/04 protocol treatment. MODS refers to ‘the development of potentially reversible physiologic derangement affecting two or more organ systems that are not directly involved in the condition leading to intensive care unit admission, and occurring after a potentially life‐threatening physiologic insult’. The clinical factors assessed included paediatric risk of mortality score III (PRISM III), paediatric logistic organ dysfunction score‐2 (PELOD‐2).

We assessed therapeutic responses at 4 weeks and monitored dynamic responses based on response time and disease progression. Early response was defined as resolving all clinical manifestations and normalising HLH‐related laboratory findings, such as a complete blood count or other relevant parameters at 4 weeks. Patients who meet the following criteria at 4 weeks were classified as having achieved a complete response (CR): absence of fever, splenomegaly, cytopenia, hypertriglyceridemia, ferritin levels below 500 μg/L, and normal cerebral spinal fluid. A partial response (PR) was generally defined as an improvement of at least ≥ 2 symptoms and laboratory markers after initiation of treatment. No response (NR) was considered a treatment failure and/or 50% worsening in two or more signs or laboratory abnormalities within 4 weeks [7].

## 3 Statistical Analysis

The data was summarised using descriptive statistics, including medians, ranges, and standard deviations or standard errors. The Mann–Whitney *U* test or chi‐square test was employed to compare differences between the two groups based on variable categories. Univariate and multivariate Cox proportional hazards models were used to determine the correlations among clinical data, laboratory variables and outcomes. OS was measured as the time from HLH diagnosis to the date of death from any cause or the last follow‐up.

Causal mediation analysis is a methodology that enables researchers to evaluate both direct and indirect natural effects of various variables [8]. It allows us to shift our focus from determining the effectiveness of a treatment to understanding the mechanism of action [9]. By utilising mediators, such as early responses (CR at 4 weeks), causal mediation analysis can elucidate the mechanism by which the intervention (in this study, different treatments) influences the outcome (in this study, OS) [10]. Therefore, it is crucial to determine whether reaching complete remission at 4 weeks could serve as a mediating factor in a causal mediation analysis for an adaptive therapy approach. The total effect of the treatment comprises two parts: the indirect effect and the direct effect. This study evaluated CR as the mediator at 4 weeks. The baseline covariates in mediation analysis included EBV infection, central nervous system‐HLH (CNS‐HLH), splenomegaly, paediatric intensive care unit (PICU) admission, ferritin, lactate dehydrogenase (LDH), albumin (ALB), aspartate transferase (AST), total bilirubin (TBIL), and direct bilirubin (DBIL). Mediation analysis was employed to evaluate the direct, indirect, and total effects of different therapies on survival outcomes [11, 12]. All statistical analyses were performed using SPSS version 26.0 (IBM, Armonk, NY) and R version 4.0.2. Statistical significance was defined as a two‐tailed *p* value < 0.05.

## 4 Results

### 4.1 Patient Demographics and Laboratory Characteristics

**FIGURE 1:**
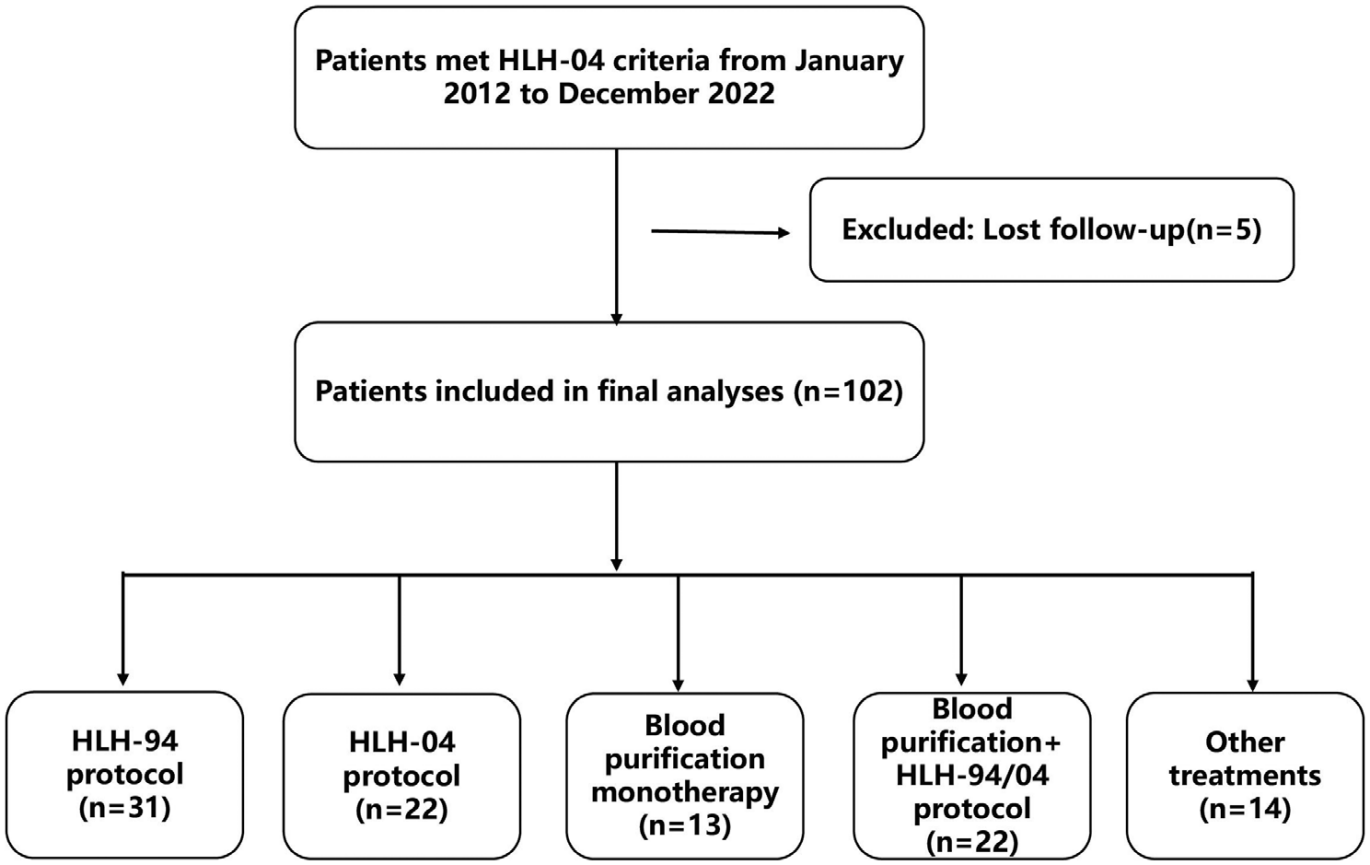
The flow chart displays the distribution of enrolled patients.

This study enrolled 102 patients in total. Of these, 31 were treated according to the HLH‐94 protocol, and 22 followed the HLH‐04 protocol (see Figure 1). Additionally, 13 patients received BP monotherapy, while 22 were treated with BP in combination with the HLH‐94/04 protocol. Of the 35 patients, 6 received plasmapheresis alone, while the remaining 29 underwent both plasmapheresis alone and plasmapheresis combined with continuous venovenous hemofiltration (CVVH). The male‐to‐female ratio in the study was 1.55 (62:40), as shown in Table 1. Five patients were diagnosed with primary HLH. These mutations included X‐linked lymphoproliferative Disease Type 1 (XLP1), X‐linked lymphoproliferative syndrome‐2 (XLP‐2), Magnesium transporter 1 (MAGT1), Unc‐13 Homologue D (UNC13D), and SH2 Domain Containing 1A (SH2D1A).

**TABLE 1:** Demographic and clinical characteristics among patients with HLH.

	All (*n* = 102)	HLH‐94 protocol treatment (*n* = 31)	HLH‐04 protocol treatment (*n* = 22)	Blood purification monotherapy (*n* = 13)	Blood purification and HLH‐94/04 protocol (*n* = 22)	Other treatments^a^ (*n* = 14)	*p*
Gender, male, *n* (%)	62 (60.8)	20 (64.5)	10 (45.5)	9 (69.2)	13 (59.1)	10 (71.4)	0.488
Age, years, median (range)	3 (0.2, 18)	3 (0.4, 15)	2 (0.8, 13)	3 (0.3, 8)	3 (0.2, 9)	2 (0.2, 18)	0.465
Primary HLH, *n* (%)	5 (4.9)	3 (9.7)	—	—	2 (9.1)	—	0.394
Secondary HLH							
EBV infection, and (%)	68 (66.7)	23 (74.2)	15 (68.2)	6 (46.2)	16 (72.2)	8 (57.1)	0.378
Other infections, and (%)	6 (5.9)	1 (3.2)	—	2 (15.4)	2 (9.1)	1 (7.1)	0.362
Malignancies, *n* (%)	2 (2.0)	1 (3.2)	—	—	1 (4.5)	—	0.735
Autoimmune diseases, *n* (%)	4 (3.9)	1 (3.2)	1 (4.5)	1 (7.7)	—	1 (7.1)	0.766
Unknown, *n* (%)	17 (16.7)	2 (6.5)	6 (27.3)	4 (30.8)	1 (4.5)	4 (28.6)	**0.045** *
HLH‐04 criteria							
Fever, *n* (%)	100 (98.0)	31 (100)	20 (90.9)	13 (100)	22 (100)	14 (100)	0.115
Splenomegaly, *n* (%)	69 (67.8)	23 (74.2)	16 (72.7)	9 (69.2)	13 (59.1)	8 (57.1)	0.677
Bicytopenia, *n* (%)	76 (74.5)	23 (74.2)	17 (77.3)	9 (69.2)	16 (72.7)	11 (78.6)	0.979
Hypertriglyceridemia and/or hypofibrinogenemia, *n* (%)	82 (80.4)	24 (80.0)	18 (81.8)	12 (92.3)	17 (81.1)	11 (78.6)	0.887
Ferritin, μg/L, median (range)	16,826 (568, 177,730)	8178 (568, 73,435)	12,748 (906, 90,309)	11,335 (1324, 83,027)	25,970 (1846, 177,730)	37191.5 (1734, 80,287)	**0.007** *
Hemophagocytosis, *n* (%)	93 (91.8)	31 (100)	19 (86.4)	13 (100)	19 (100)	11 (78.6)	0.082
H score, median (range)	270 (141,319)	257 (171,319)	268 (141,319)	269 (174,319)	289 (239,319)	266 (244,304)	0.109
CNS involvement, *n* (%)	13 (12.8)	2 (6.5)	1 (4.5)	2 (15.4)	8 (36.4)	—	**0.004** *
Length of onset to diagnosis, days, median (range)	11 (1, 48)	11 (5, 41)	14 (3, 48)	11 (4, 42)	8.5 (2, 35)	12 (1, 32)	0.128
PICU, *n* (%)	79 (76.5)	21 (67.7)	14 (63.6)	13 (100)	22 (100)	9 (64.3)	**0.012** *
Ventilatory support, *n* (%)	40 (39.2)	5 (16.1)	3 (13.6)	12 (92.3)	13 (59.0)	7 (50.0)	**< 0.001** *
Length of PICU, days, median (range)	8 (0, 49)	3 (0, 37)	4.5 (0, 26)	9 (0, 18)	14 (0, 49)	3.5 (0, 33)	**0.002** *
Assessment of response, *n* (%)							
CR at 4 weeks	59 (57.8)	24 (77.4)	19 (86.4)	2 (15.4)	8 (36.4)	6 (42.9)	**< 0.001** *

*Note:* Bold values indicates statistically significant differences, highlighting meaningful statistical relevance.

Abbreviations: CNS, central nervous system; CR, complete response; EBV, Epstein–Barr virus; HLH, hemophagocytic lymphohistiocytosis; PICU, paediatric intensive care unit.

^a^ Other treatments include corticosteroids, intravenous immunoglobulin therapy, and/or ruxolitinib therapy.

* *p* < 0.05.

The HLH‐04 protocol group had a superior CR rate of 86.4% after 4 weeks, in contrast to the HLH‐94 protocol, which achieved a CR rate of 77.4%. The BP monotherapy and the BP combined with HLH‐94/04 had lower CR rates at 4 weeks: 15.4% and 36.4%, respectively. Patients in these groups also showed higher levels of central infiltration, ferritin, and bilirubin than the HLH‐94/04 protocol group (Table S1). They also had higher rates of treatment with ventilatory support in the paediatric intensive care unit (PICU) and longer PICU stays (*p* < 0.05).

A matched subgroup analysis was conducted in patients with MODS to compare baseline clinical characteristics and assess survival differences among treatment groups (Table S2). All patients required ventilatory support. Median PELOD‐2 scores were significantly higher in the BP monotherapy group (*p* = 0.049), indicating greater organ dysfunction. CR at 4 weeks was more frequent in the HLH‐94/04 group (50%) compared to BP monotherapy (8.3%) and BP combined with HLH‐94/04 (15.4%) (*p* = 0.072).

**FIGURE 2:**
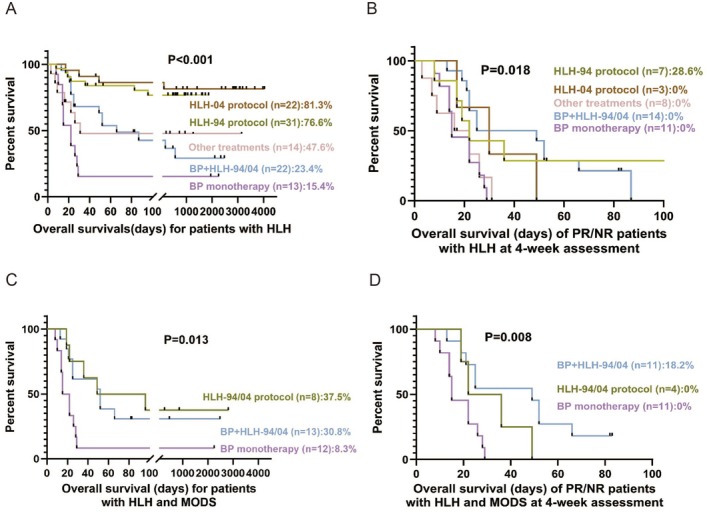
Survival outcomes in children with HLH. (A) Overall survival of patients with HLH receiving different treatments. (B) Survival of patients with partial or no response (PR/NR) at the 4‐week assessment. (C) Survival of patients with HLH and multiple organ dysfunction syndrome (MODS) under various treatments. (D) Survival of HLH patients with MODS assessed as PR/NR at the 4‐week assessment.

The OS of patients with HLH differed significantly by various treatments (Figure 2). Patients treated with the HLH‐04 and HLH‐94 protocols demonstrated the highest survival rates (81.3% and 76.6%, respectively), compared to those receiving other treatments (47.6%), BP combined with HLH‐94/04 (23.4%), or BP monotherapy (15.4%) (*p* < 0.001). A subgroup analysis of PR/NR patients with HLH at the 4‐week assessment, only the HLH‐94 protocol showed a survival advantage (28.6%) (*p* = 0.018). In patients with HLH complicated by MODS, survival was highest with the HLH‐94/04 protocol at 37.5%, but this was not significantly different from the 30.8% survival rate observed with BP combined with the HLH‐94/04 (*p* = 0.667). Notably, in PR/NR patients with both HLH and MODS, only the BP combined with HLH‐94/04 showed any survival (18.2%) (*p* = 0.008).

### 4.2 Prognostic Factors Analysed Using Cox Regression

According to the results of the univariate Cox regression analysis, several risk factors contributed to overall mortality in HLH. These factors include EBV infection, splenomegaly, CNS involvement, admission to the PICU, elevated levels of ferritin, AST, LDH, TBIL, DBIL, BP monotherapy, BP combined with HLH‐94/04, other treatments, and CR at the 4‐week assessment (refer to Table S3).

Multiple Cox analyses found that patients with HLH who had CNS involvement, high LDH levels, and PR/NR at the 4‐week assessment had a higher risk of mortality (Figure S1) (*p* < 0.05). Compared to the HLH‐94 protocol treatment, it was observed that the BP monotherapy and other treatments were associated with an increased risk of death. Furthermore, the BP combined with HLH‐94/04 reduced the risk of death, although this finding was not statistically significant.

### 4.3 Causal Mediation Analysis

**FIGURE 3:**
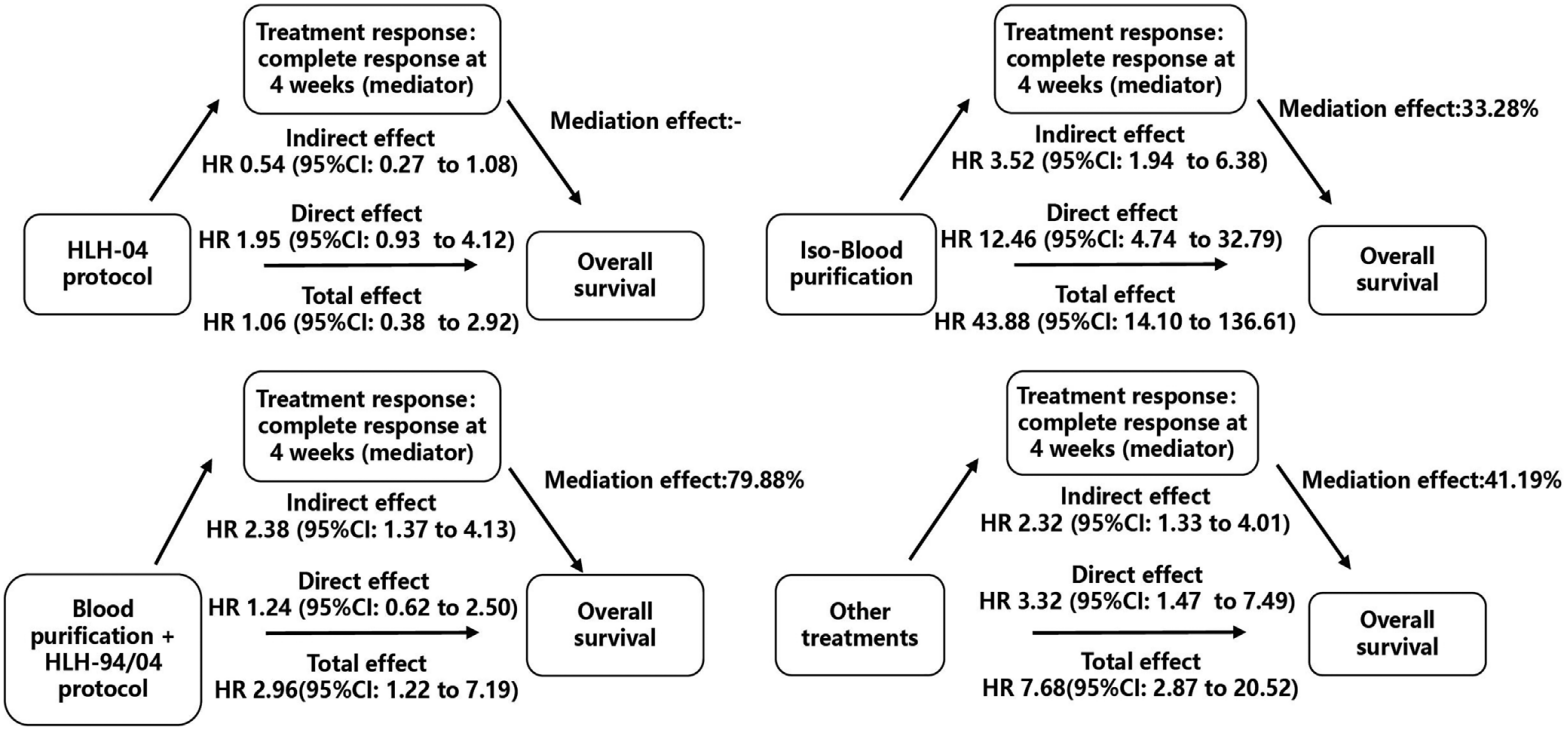
Directed acyclic graph illustrating the effect of treatment response at 4 weeks on overall survival (OS), relative to the HLH‐94 protocol.

Patients receiving different treatments showed variable early responses and OS. To further evaluate this relationship, we assessed the effect of achieving CR at the 4‐week mark on OS across treatment groups (Figure 3). The HLH‐94 protocol served as the reference. The HLH‐04 protocol achieved CR at 4 weeks and had a comparable impact on OS to HLH‐94 (*p* > 0.05). In contrast, BP monotherapy achieving CR at 4 weeks had a limited effect on OS, contributing only 33.28%. Notably, patients receiving BP combined with HLH‐94/04 who achieved CR at 4 weeks demonstrated a substantially higher effect on OS (79.88%). These findings suggest that CR achieved through BP monotherapy has a limited influence on long‐term survival and is associated with poorer prognosis, whereas CR achieved through combination therapy with HLH‐94/04 confers a survival benefit similar to that of the HLH‐94 protocol.

## 5 Discussion

HLH is a life‐threatening syndrome characterised by excessive inflammation and dysregulated immune activation [13]. Extracorporeal BP has been shown to effectively remove inflammatory cytokines and provide organ support [14]. Although current clinical evidence for BP in HLH remains limited and is largely extrapolated from sepsis and MODS contexts [3, 15, 16], our study provides novel, subgroup‐specific data supporting its adjunctive role. In subgroup analyses of patients classified as partial or non‐responders (PR/NR) and those with MODS, only the group treated with BP combined with the HLH‐94/04 showed survival at 4 weeks. This observation suggests a potential synergistic effect when BP is integrated with HLH‐directed therapy, particularly in patients with poor early responses or severe organ dysfunction.

BP monotherapy was associated with poor outcomes, especially in PR/NR patients and those with MODS, suggesting it is insufficient as a standalone treatment for HLH. HLH is driven by uncontrolled immune activation and cytokine storms, which cannot be effectively managed by cytokine removal alone. In our study, BP referred to plasmapheresis alone or combined with CVVH, both of which have limited cytokine clearance compared to hemoadsorption or high‐volume plasma exchange [17]. These technical limitations may partly explain the limited benefit of BP monotherapy. Therefore, BP should be considered a supportive adjunct, not a replacement for standard immunochemotherapy.

Our causal mediation analysis revealed that the modest survival benefit seen in the BP combined with the HLH‐94/04 may be partly explained by the high proportion of patients with MODS in this group. Additionally, potential adverse effects of BP—such as coagulation abnormalities and fluctuations in drug concentrations—could counteract its intended therapeutic effects [18]. Despite this, BP may offer particular benefit in HLH patients with liver failure, where it can support hepatic detoxification and inflammatory control [19]. Therefore, BP should be considered a supportive measure in patients unresponsive to HLH‐94/04 or in those who develop progressive organ dysfunction [20]. Further investigation into its timing, safety, and efficacy in combination with standard treatment is warranted.

Our data also reinforce the critical prognostic value of early treatment response. Achieving CR at 4 weeks was associated with significantly better survival outcomes, suggesting that early response is a more reliable mediator of OS than later timepoints. This aligns with previous studies indicating that early treatment response is one of the strongest predictors of long‐term outcomes in HLH [21, 22]. Current protocols, such as the TPOG NHL guideline, recommend intensifying therapy or proceeding to haematopoietic stem cell transplantation (HSCT) in patients with inadequate response by week 4 [23]. Furthermore, recent studies suggest that response by day 7 may be the most accurate predictor of pre‐transplant mortality, supporting early, response‐adapted treatment strategies in HLH [24]. Early assessment may thus serve both as a prognostic indicator and as a substitute endpoint for guiding therapeutic adjustments [25, 26].

## 6 Conclusion

HLH‐94/04 remains the cornerstone of paediatric HLH treatment. Adding BP may improve survival in patients with MODS or poor early response, but BP alone is ineffective. Achieving CR at 4 weeks is a key prognostic marker and should guide early therapeutic decisions. Further studies are needed to confirm this benefit and determine optimal timing, modality and patient selection.

## Author Contributions

**Lihua Yu:** data curation (lead), formal analysis (lead), investigation (lead), methodology (lead), software (lead), supervision (equal), writing – original draft (lead), writing – review and editing (lead). **Danna Lin:** conceptualization (lead), data curation (lead), resources (equal), software (equal), visualization (equal), writing – original draft (equal), writing – review and editing (equal). **Li Wu:** conceptualization (equal), formal analysis (lead), resources (equal), writing – original draft (lead), writing – review and editing (equal). **Lulu Huang:** software (equal), supervision (equal), validation (equal). **Xiaorong Lai:** conceptualization (equal), formal analysis (equal), investigation (equal), methodology (equal). **Yajie Zhang:** data curation (equal), formal analysis (equal), supervision (equal), validation (equal). **Juan Zi:** data curation (equal), funding acquisition (equal), investigation (equal), methodology (equal). **Jingxin Zhang:** conceptualization (equal), funding acquisition (equal), writing – original draft (equal), writing – review and editing (equal). **Xu Liao:** formal analysis (equal), funding acquisition (equal), investigation (equal), methodology (equal). **Lichan Liang:** conceptualization (equal), data curation (equal), formal analysis (equal), validation (equal), visualization (equal), writing – original draft (equal), writing – review and editing (equal). **Guanmei Zhang:** data curation (equal), software (equal), supervision (equal), validation (equal), visualization (equal). **Liucheng Yang:** conceptualization (equal), data curation (equal), formal analysis (equal), funding acquisition (equal), software (equal), supervision (equal). **Lihua Yang:** conceptualization (lead), funding acquisition (lead), project administration (lead), resources (lead), software (lead), writing – review and editing (lead).

## Ethics Statement

The study titled ‘Retrospective Analysis of Treatment Outcomes and Prognostic Factors in Children with Hemophagocytic Lymphohistiocytosis’ was approved by the Institutional Review Board of Zhujiang Hospital of Southern Medical University on December 1, 2022 (ID:2023‐KY‐258‐01).

## Consent

Written informed consent from the participants' legal guardian/next of kin was not required to participate in this study in accordance with the national legislation and the institutional requirements.

## Conflicts of Interest

The authors declare no conflicts of interest.

## Supporting information


**Table S1:** Laboratory data among patients with HLH.
**Table S2:** Hemophagocytic lymphohistiocytosis‐associated with MODS.
**Table S3:** Risk factors for survival by univariate Cox regression.
**Figure S1:** The risk factors affecting overall survival were analysed using multivariate Cox regression to assess the different parameters.

## Data Availability

The datasets utilised and/or analysed during the current study can be obtained from the corresponding author upon reasonable request. Requests may be sent to: yanglihua@smu.edu.cn.
