# Supplementary material for: Effect of Blood Purification on Early Response in Children With Hemophagocytic Lymphohistiocytosis
Source: J Cell Mol Med. 2025 Nov 3;29(21):e70811. doi: 10.1111/jcmm.70811 (PMC12580619; doi:10.1111/jcmm.70811)
Supplement: Supplementary file 1 — Table S1: Laboratory data among patients with HLH. Table S2: Hemophagocytic lymphohistiocytosis‐associated with MODS. Table S3: Risk factors for survival by univariate Cox regression. Figure S1: The risk factors affecting overall survival were analysed using multivariate Cox regression to assess the different parameters. [file JCMM-29-e70811-s001.docx]

Effect of Blood Purification on Early Response in Children with Hemophagocytic Lymphohistiocytosis

Lihua Yu ^1^*, Danna Lin ^1^*, Li Wu, MD ^1^, Lulu Huang ^1^, Xiaorong Lai ^1^, Yajie Zhang ^1^, Juan Zi ^1^, Jingxin Zhang ^1^, Xu Liao ^1^, Lichan Liang ^1^, Guanmei Zhang ^1^, Liucheng Yang ^2#^, Lihua Yang ^1#^

^2^Department of Pediatric Surgery, Zhujiang Hospital, Southern Medical University, Guangzhou, Guangdong, China;

*These authors contributed equally to this work.

^#^These authors were corresponding authors and contributed equally to this work.

Correspondence to: Liucheng Yang, Department of Pediatric Surgery, Zhujiang Hospital, Southern Medical University, Guangzhou, China. E-mail: sdylc@aliyun.com; Lihua Yang. Department of Pediatric Hematology, Zhujiang Hospital, Southern Medical University, Guangzhou, China. E-mail: [dryanglihua@163.com](mailto:dryanglihua@163.com).

**Keyword**

Hemophagocytic Lymphohistiocytosis, Pediatric, Mediation Analysis, Blood Purification, Treatment Responses

| Table S1 Laboratory data among patients with HLH | | | | | | | |
| --- | --- | --- | --- | --- | --- | --- | --- |
|  | All (n=88) | HLH-94 protocol treatment (n=31) | HLH-04 protocol treatment (n=22) | Blood purification monotherapy (n=13) | Blood purification and  HLH-94/04 protocol (n=22) | Other treatments^a^ (n=14) | P value |
| WBC, ×10^9^/L, median (range) | 2.04  (0.17, 105.48) | 2.32  (0.33,15.77) | 2.06  (0.4,85.78) | 2.8  (0.41, 16.33) | 1.97  (0.17, 105.48) | 1.92 (0.64,13.38) | 0.859 |
| Neutrophil, ×10^9^/L, median (range) | 0.73  (0.00,12,54) | 0.61  (0.02,9.07) | 0.57 (0.12,11.92) | 1.58 (0.13,12.54) | 0.81  (0.00,7.28) | 0.81 (0.00,4.45) | 0.138 |
| Hemoglobin, g/L, (range) | 90.5  (30,125) | 91  (55,112) | 92  (56,112) | 92  (62,112) | 83.5  (39,125) | 82.5  (30,99) | 0.260 |
| Platelet, ×10^9^/L, median (range) | 43.5  (7,352) | 45  (9,195) | 61  (10,221) | 40  (7,352) | 34 (9,170) | 49.5  (20,170) | 0.382 |
| Triglycerides, mmol/L, median (range) | 3.48  (0.97,14.55) | 3.84  (1.07, 8.65) | 3.46  (1.72, 14.55) | 3.34  (0.97,8.39) | 3.83  (1.63,8.39) | 3.3 (1.87,7.02) | 0.705 |
| Fibrinogen, g/L, median (range) | 1.4  (0.00,6.25) | 1.5  (0.00, 3.87) | 1.8  (0.5,5.96) | 1.23  (0.43,6.25) | 1.15  (0.00,2.85) | 1.24 (0.31,3.82) | 0.431 |
| ALT, U/L, median (range) | 128.5  (5,3534) | 91  (5,720) | 118  (10,529) | 121  (9.1,3534) | 152  (39,501) | 152  (19,446) | 0.703 |
| AST, U/L, median (range) | 312.5  (18,9452) | 151  (24,819) | 323  (18,847) | 380  (24,9452) | 488  (91,2307) | 477.5 (21,1688) | **0.006*** |
| LDH, U/L, median (range) | 1700  (190.30,15544) | 1663.65  (190.30,15544) | 1575 (302.5,11230) | 1420.5 (375,5840) | 2346.7 (884.1,13044.00) | 2395.25 (381.8,6510) | **0.011*** |
| TBIL, µmol/L, median (range) | 25.3  (4.30,167.3) | 18.35 (4.3,130.90) | 19.25  (5.1,90.1) | 57.9  (6.0,102.6) | 60  (8.1,167.3) | 22 (7.6,131.5) | **0.002*** |
| DBIL, µmol/L, median (range) | 17.6  (0.5,110.1) | 12.85 (0.5,102.8) | 11.5  (1.10,66.7) | 39.9  (4.9,84.3) | 48.6  (6.0,110.10) | 13.1 (3.3,87.7) | **<0.001*** |
| ALB, g/L, median (range) | 27.7  (11.9,43.9) | 28.35  (11.90,43.90) | 30.4 (24.3,41.40) | 25.3 (18.70,39.20) | 26.7  (20.7,40.6) | 26.8 (14.7,37.1) | 0.168 |
| Abbreviations: HLH, hemophagocytic lymphohistiocytosis; WBC, white blood count; ALT, alanine transaminase; AST, aspartate transferase; LDH, lactate dehydrogenase; TBIL, total bilirubin; DBIL, direct bilirubin; ALB, albumin. * p < 0.05. | | | | | | | |

| Table S2 Hemophagocytic lymphohistiocytosis-associated with MODS | | | | |
| --- | --- | --- | --- | --- |
|  | HLH-94/04 protocol treatment (n=8) | Blood purification monotherapy  (n=12) | Blood purification and HLH-94/04 protocol (n=13) | P value |
| Ferritin, μg/L, median (range) | 15212 (928,62655) | 14071 (1324,83027) | 30894 (5241，177730) | 0.178 |
| CNS involvement, (n %) | 2 (25.0%) | 2 (16.7%) | 3 (23.1%) | 0.888 |
| Ventilatory Support, n (%) | 8 (100.0%) | 12 (100.0%) | 13 (100.0%) | 1.000 |
| Vasoactive agents, n (%) | 4 (50.0%) | 10 (83.3%) | 11 (84.6%) | 0.157 |
| Plasmapheresis | - | 3 (25.0%) | 2 (15.4%) | 0.498 |
| Plasmapheresis+CVVH | - | 9 (75.0%) | 11 (84.6%) | 0.498 |
| PELOD-2 | 8 (4,10) | 11.5 (7,18) | 9 (4,18) | **0.049*** |
| PRISM III | 19 (13,22) | 28 (8,38) | 27 (10,37) | 0.061 |
| CR at 4 weeks | 4 (50.0%) | 1 (8.3%) | 2 (15.4%) | 0.072 |
| Abbreviations: MODS: multi-organ dysfunction syndrome; HLH: hemophagocytic lymphohistiocytosis; CNS, Central nervous system; CVVH: continuous venovenous hemofiltration; PELOD-2: pediatric logistic organ dysfunction score-2; PRISM III: pediatric risk of mortality score III; CR: complete response. * p < 0.05. | | | | |

| Table S3 Risk factors for survival by univariate Cox regression | | |
| --- | --- | --- |
| Variables | HR (95% CI) | P value |
| Age | 0.917 (0.822,1.022) | 0.118 |
| EBV infection | 2.742 (1.276,5.893) | **0.010*** |
| Fever | 20.996 (0.006,67883) | 0.461 |
| Splenomegaly | 0.481 (0.266,0.867) | **0.015*** |
| Bicytopenia | 1.607 (0.773,3.338) | 0.204 |
| Hypertriglyceridemia and/or hypofibrinogenemia | 1.657 (0.700,3.923) | 0.250 |
| Ferritin | 1.000 (1.000,1.000) | **0.017*** |
| Hemophagocytosis | 2.131 (0.293,15.489) | 0.455 |
| CNS involvement | 2.464 (1.246,4.874) | **0.01*** |
| Length of onset to diagnosis | 0.974 (0.935,1.013) | 0.189 |
| PICU | 4.189 (1.498,11.714) | **0.006*** |
| Length of PICU | 1.018 (0.995,1.042) | 0.133 |
| WBC | 0.959 (0.895,1.028) | 0.238 |
| Neutrophil | 0.954 (0.831,1.096) | 0.507 |
| Hemoglobin | 0.981 (0.961,1.001) | 0.063 |
| Platelet | 0.993 (0.984,1.001) | 0.09 |
| Triglycerides | 0.983 (0.858,1.126) | 0.8 |
| Fibrinogen | 0.811 (0.599,1.096) | 0.173 |
| ALT | 1.001 (1.000,1.001) | 0.134 |
| AST | 1.000 (1.000,1.001) | **0.002*** |
| LDH | 1.000(1.000,1.000) | **0.007*** |
| TBIL | 1.011 (1.003,1.019) | **0.006*** |
| DBIL | 1.013 (1.003,1.023) | **0.009*** |
| ALB | 0.932 (0.884,0.982) | **0.009*** |
| HLH-94 protocol treatment | 0.34 (0.152,0.763) | **0.009*** |
| HLH-04 protocol treatment | 0.265 (0.095,0.741) | **0.011*** |
| Blood purification monotherapy | 4.480 (2.230,9.000) | **<0.001*** |
| Blood purification and HLH-94/04 protocol | 2.036 (1.093,3.794) | **0.025*** |
| Other treatments | 1.533 (0.684,3.436) | 0.299 |
| CR at 4 weeks | 0.019 (0.007,0.053) | **<0.001*** |
| Abbreviations: HLH, hemophagocytic lymphohistiocytosis; EBV, Epstein-Barr virus; CNS: Central nervous system WBC, white blood count; ALT, alanine transaminase; AST, aspartate transferase; LDH, lactate dehydrogenase; TBIL, total bilirubin; DBIL, direct bilirubin; ALB, albumin; PICU: Pediatric Intensive Care Unit. | | |

、


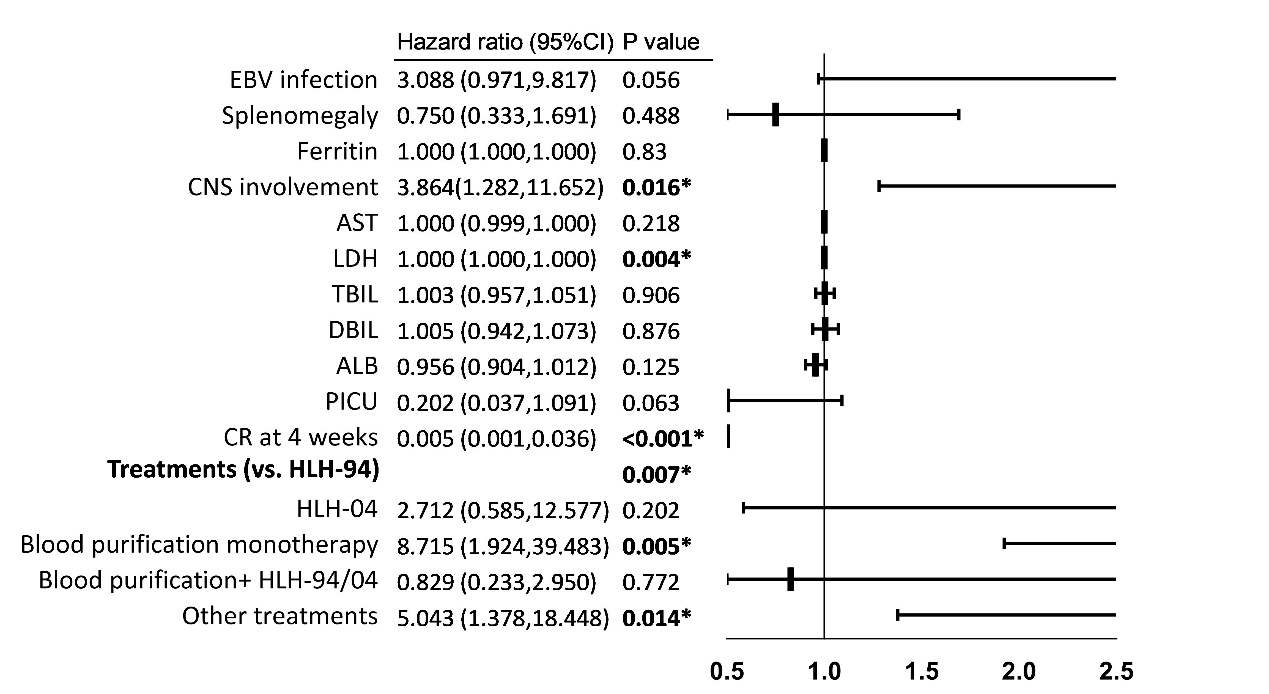


Figure S1. The risk factors affecting overall survival were analyzed using multivariate Cox regression to assess the different parameters.
